# Haplogroup Context is Less Important in the Penetrance of Mitochondrial DNA Complex I Mutations Compared to mt-tRNA Mutations

**DOI:** 10.1007/s00239-018-9855-7

**Published:** 2018-07-09

**Authors:** Hannah O’Keefe, Rachel A. Queen, Surita Meldau, Phillip Lord, Joanna L. Elson

**Affiliations:** 1 Institute of Genetic Medicine, Newcastle University, Newcastle-upon-Tyne, NE1 3BZ UK; 2 Inherited Metabolic Disease Laboratory, National Health Laboratory Services, Cape Town, South Africa; 3 School of Computing, Newcastle University, Newcastle-upon-Tyne, NE4 5TG UK; 4 Division of Chemical Pathology, University of Cape Town, Cape Town, South Africa; 5 Centre for Human Metabonomics, North-West University, Potchefstroom, South Africa

**Keywords:** Mitochondria, Complex I, Pathogenicity, Variants, Sequence context, Haplogroups

## Abstract

Mitochondrial diseases are a highly complex, heterogeneous group of disorders. Mitochondrial DNA variants that are linked to disease can exhibit variable expression and penetrance. This has an implication for mitochondrial diagnostics as variants that cause disease in one individual may not in another. It has been suggested that the sequence context in which a variant arises could influence the genotype–phenotype relationship. However, the consequence of sequence variation between different haplogroups on the expression of disease is not well understood. European haplogroups are the most widely studied. To ensure accurate diagnostics for patients globally, we first need to understand how, if at all, the sequence context in which a variant arises contributes to the manifestion of disease. To help us understand this, we used 2752 sequences from 33 non-human species that do not have disease. We searched for variants in the seven complex I genes that are associated with disease in humans. Our findings indicate that only three reported pathogenic complex I variants have arisen in these species. More importantly, only one of these, m.3308T>C, has arisen with its associated amino acid change in the studied non-human species. With the status of m.3308T>C as a disease causing variant being a matter of debate. This is a stark contrast to previous findings in the mitochondrial tRNA genes and suggests that sequence context may be less important in the complex I genes. This information will help us improve the identification and diagnosis of mitochondrial DNA variants in non-European populations.

**Electronic supplementary material:**

The online version of this article (10.1007/s00239-018-9855-7) contains supplementary material, which is available to authorized users.

## Introduction

Diseases resulting from mitochondrial DNA (mtDNA) mutations are a clinically heterogeneous group of disorders frequently exhibiting variable penetrance. The prevalence of mitochondrial disease is estimated to be approximately 1 in 4300 within the adult European population (Gorman et al. 2015). Assessment of pathogenicity is difficult, requiring evidence from multiple sources to obtain reliable information to link a mutation to disease. These difficulties are in part due to high levels of mtDNA variation within any given population (van Oven and Kayser 2009) and lack of standards in the use of evidence to link variants to disease (Yarham et al. 2012). European populations remain the most widely studied in regards to mitochondrial disease. Other populations, such as Black Africans, have been studied far less and the impact of mitochondrial variants within these populations is not yet known (van der Westhuizen et al. 2015). It is debated whether haplogroup background influences the penetrance of mtDNA mutations. Some populations may be more or less susceptible to the pathogenic effect of particular mtDNA variants (Ji et al. 2014).

This may partially explain the low diagnosis rates seen within non-European populations where population variation is still less explored. Studying haplogroup background and its impact on disease penetrance will help ensure similar levels of diagnostic accuracy to all populations (van der Walt et al. 2012; van der Westhuizen et al. 2015). One way to begin to achieve this increased understanding of the importance of haplogroup context is by searching for variants, which are known to be pathogenic in humans, in other species. Magalhães (2005), conducted a search using a single mitochondrial genome sequence in each of 12 *primate* species. In total 46 disease-associated variants were found to be present in 1 or more of the *primates*. Magalhães (2005) suggests that masking variants may exist which could nullify the deleterious effects of pathogenic variants. This supports the theory that haplogroup background is important in the expression and penetrance of disease in humans. The repertoire of disease-associated variants has expanded since the publication of this article and clinically validated scoring criteria are now available for the assessment of pathogenicity. Similarly, a wealth of publicly available sequences, both human and non-human, are now available. Recently, Queen et al. (2017) used a significantly expanded collection of sequences from multiple chordate species and successfully identified human pathogenic variants and possible masking variants in mitochondrial tRNA’s (mt-tRNA’s), particularly in mt-tRNA-Leu (UUR). In the current paper, pathogenic variants of the complex I mtDNA encoded subunits were searched for in a collection of 2752 sequences from 33 non-human species as described previously (2017). We identified a single variant, m.3308T>C, that is both reported as pathogenic in humans (Mezghani et al. 2013; Ding and Zhu 2011; Zarrouk Mahjoub et al. 2012) and results in an amino acid change in the mtDNA context of the species studied. However the strength of the evidence to support the pathogenicity of this variant has been debated (Salas and Elson 2012). This scarcity of disease-associated protein-coding variants seen in other species and debate over the pathogenicity of those that have been observed is in sharp contrast to what was described in the context of the mt-tRNA’s where many more pathogenic variants were seen (Queen et al. 2017).

## Methods

### Identification of Disease-Associated Variants

The MitoMap database was utilised to identify nucleotide variants which have been previously associated with disease. All nucleotide variants from genes which encode mitochondrial complex I were collated. (Accessed:02-2017).

### Extraction of the Revised Cambridge Reference Sequence from the NCBI GenBank Database

The revised Cambridge Reference Sequences (rCRS) for human mitochondrial *ND1-6* and *ND4L* were obtained from the NCBI GenBank database, NCBI Ref Seq: NC_012920.1. The corresponding gene from the rCRS was inserted into FASTA files of individual gene sequences from non-human species. FASTA files were pre-compiled by Queen et al. (2017), as described previously, see Supplementary Table 1.

### Gene Alignment and Variant Calling

Species sequence files with the inserted rCRS were uploaded into Jalview software (Waterhouse et al. 2009) for alignment. Clustal Omega alignments were performed with default settings. Nucleotide positions identified through MitoMap were located in each sequence alignment and assessed for variability from the rCRS.

### Pathogenicity Scoring and Assessing the Pathogenicity of Amino Acid Substitutions

Pathogenicity scoring was performed on nucleotide variants identified in the alignments of non-human species. This was conducted in accordance with the Mitchell et al. (2006) pathogenicity scoring algorithm (Mitchell et al. 2006). Human amino acid reference sequences were identified in UniProt as follows: P03886, P03891, P03897, P03905, P03915, P03923, P03901. PolyPhen-v2 and MutPred1.0 were consulted for amino acid pathogenicity predictions as described previously.

### Potential Masking Variants and their Presence as Population Markers in Human Haplogroups

Alignments were screened for additional variants which may mask the deleterious effects of the pathogenic variants. Putative masking variants were also scored for pathogenicity. MitoMap and NCBI GenBank were used to search for the putative masking variants within populations.

## Results

### Classification of Variants Conserved in Non-human Species

Of the 152 complex I variants initially identified as being human disease-associated through the MitoMap database (Lott et al. 2013a), 87 were found in the complex I genes of the non-human species studied here. 11 of these would result in synonymous amino acids changes and were not investigated further. Pathogenicity was fully assessed for each of the remaining 76 variants with the use of the Mitchell et al. (2006) pathogenicity scoring algorithm, as well as PolyPhen v2 (Adzhubei et al. 2010) and MutPred1.0 (Li et al. 2009) mutation prediction software. Based on this assessment, three variants were classified as definitely pathogenic; 19 as probably pathogenic (Table 1); 26 as possibly pathogenic and 28 as neutral or polymorphic variants, see Supplementary Table 2. Additional data relating to GenBank frequency and conservation index for each variant listed were obtained using the 45,494 full length sequences included in the SNV query tool of Mitomaster (Lott et al. 2013b) (Table 2).

**Table 1:** Definitely or Probably pathogenic human mitochondrial variants present in the alignments of one or more chordate species across all 7 complex I genes

Gene	Position	Variant	Disease association	Status	Species
ND1	3308	T-C	MELAS, SIDS, putative LHON	Definitely pathogenic	*Pan paniscus, Pan troglodytes troglodytes, Pan troglodytes schweinfurthii, Pan troglodytes verus*
	3310	C-T	Diabetes, hypertrophic cardiomyopathy	Probably pathogenic	*Mus musculus, Mus musculus domesticus, Rattus norvegicus, Myodes glareolus, Bos taurus, Bos grunniens, Ovis aries, Balaenoptera physalus, Bison bison, Orcinus orca, Sus scrofa, Syncerus caffer, Tursiops truncatus, Canis lupus familiaris, Urocyon littoralis catalinae, Urocyon littoralis clemente, Urocyon littoralis santacruzae, Ursus arctos, Ursus spelaeus*
	3394	T-C	LHON, diabetes	Probably pathogenic	*Macaca fascicularis*
	3890	G-A	Progressive encephalomyopathy, LS, optic atrophy	Probably pathogenic	*Sus scrofa*
	3995	A-G	MELAS	Probably pathogenic	*Canis lupus familiaris*
	4171	C-A	LHON	Probably pathogenic	*Anguilla anguilla, Anguilla rostrata, Sus scrofa*
	4216	T-C	LHON, insulin resistance	Probably pathogenic	*Mus musculus, Mus musculus domesticus, Myodes glareolus, Bos taurus, Bos grunniens, Ovis aries, Equus caballus, Hypophthalmichthys molitrix, Hypophthalmichthys nobilis, Balaenoptera physalus, Bison bison, Orcinus orca, Sus scrofa, Syncerus caffer, Tursiops truncatus, Canis lupus familiaris, Urocyon littoralis catalinae, Urocyon littoralis clemente, Urocyon littoralis santacruzae, Ursus arctos, Ursus spelaeus*
ND2	4640	C-A	LHON	Probably pathogenic	*Mus musculus, Mus musculus domesticus, Rattus norvegicus, Myodes glareolus, Anguilla anguilla, Anguilla rostrata, Coregonus lavaretus, Glyphis glyphis, Hypophthalmichthys molitrix, Hypophthalmichthys nobilis, Sus scrofa, Canis lupus familiaris, Ursus arctos, Ursus spelaeus*
ND3	10,158	T-C	Leigh disease	Probably pathogenic	*Anguilla anguilla, Clupea harengus*
	10,191	T-C	Leigh disease	Definitely pathogenic	*Hypophthalmichthys molitrix*
ND4	11,240	C-T	Leigh disease	Probably pathogenic	*Rattus norvegicus, Bos taurus, Ovis aries, Equus caballus, Hypophthalmichthys nobilis, Bison bison*
	11,696	G-A	LHON, LHON with dystonia, maternally inherited deafness	Probably pathogenic	*Pan troglodytes troglodytes, Pan troglodytes schweinfurthii, Pan troglodytes verus, Macaca fascicularis, Mus musculus, Mus musculus domesticus, Rattus norvegicus, Myodes glareolus, Anguilla rostrata, Bos taurus, Ovis aries, Coregonus lavaretus, Hypophthalmichthys molitrix, Hypophthalmichthys nobilis, Balaenoptera physalus, Bison bison, Orcinus orca, Sus scrofa, Syncerus caffer, Urocyon littoralis catalinae, Urocyon littoralis clemente, Urocyon littoralis santacruzae, Ursus arctos, Ursus spelaeus*
ND4L	10,663	T-C	LHON	Probably pathogenic	*Anguilla anguilla, Anguilla rostrata, Clupea harengus, Coregonus lavaretus, Gallus gallus, Glyphis glyphis, Hypophthalmichthys molitrix, Hypophthalmichthys nobilis, Ovis aries*
ND5	13,514	A-G	Leigh disease, MELAS	Probably pathogenic	*Clupea harengus*
	13,528	A-G	LHON, MELAS	Probably pathogenic	*Macaca fascicularis, Mus musculus, Rattus norvegicus, Myodes glareolus, Anguilla anguilla, Bos grunniens, Clupea harengus, Hypophthalmichthys molitrix, Hypophthalmichthys nobilis, Balaenoptera physalus, Bison bison, Sus scrofa, Syncerus caffer, Tursiops truncatus*
	13,708	G-A	LHON, increased MS risk	Probably pathogenic	*Pan paniscus, Pan troglodytes schweinfurthii, Pan troglodytes verus, Anguilla anguilla, Anguilla rostrata, Coregonus lavaretus, Hypophthalmichthys molitrix, Hypophthalmichthys nobilis, Urocyon littoralis clemente*
ND6	14,453	G-A	Leigh disease, MELAS	Probably pathogenic	*Anguilla anguilla, Clupea harengus, Coregonus lavaretus, Gallus gallus, Glyphis glyphis, Hypophthalmichthys molitrix*
	14,459	G-A	LHON with dystonia, Leigh disease	Probably pathogenic	*Gallus gallus*
	14,482	C-A	LHON	Probably pathogenic	*Urocyon littoralis clemente, Ursus spelaeus*
	14,487	T-C	Dystonia, Leigh disease, Ataxia, Ptosis, Epilepsy	Definitely pathogenic	*Anguilla anguilla, Anguilla rostrata, Clupea harengus, Coregonus lavaretus, Glyphis glyphis, Hypophthalmichthys nobilis*
	14,502	T-C	LHON	Probably pathogenic	*Macaca fascicularis, Balaenoptera physalus, Orcinus orca*
	14,596	A-T	LHON	Probably pathogenic	*Ursus spelaeus*

Disease association is listed in accordance with reports from the Mitomap online database (Accessed: 02-2017)

**Table 2:** Human mitochondrial variants present in one or more chordate species with amino acid change, conservation index and GenBank frequency derived from MitoMaster SNV query tool (Lott et al. 2013a, b)

Gene	Position	Variant	Amino acid change	Conservation index (%)	GenBank frequency
ND1	3308	T-C	M-T	84.44	346
	3310	C-T	P-S	13.33	11
	3394	T-C	Y-H	93.33	602
	3890	G-A	R-Q	100	1
	3995	A-G	N-S	97.78	18
	4171	C-A	L-M	93.33	2
	4216	T-C	Y-H	24.44	4516
	3316	G-A	A-T	4.44	435
	3337	G-A	V-M	24.44	72
	3340	T-C	P-S	86.67	3
	3397	A-G	M-V	91.11	133
	3421	G-A	V-I	28.89	69
	3496	G-T	A-S	15.56	11
	3497	C-T	A-V	15.56	140
	3644	T-C	V-A	100	198
	3700	G-A	A-T	93.33	3
	3736	G-A	V-I	93.33	74
	3745	G-A	A-T	88.89	97
	3796	A-G	T-A	71.11	217
	3833	T-A	L-Q	40	0
	3866	T-C	I-T	86.67	127
	4132	G-A	A-T	13.33	7
	4142	G-A	R-Q	100	0
ND2	4640	C-A	I-M	26.67	132
	4648	T-C	F-S	95.56	1
	4833	A-G	T-A	17.78	428
	4917	A-G	N-D	91.11	2160
	5178	C-A	L-M	22.22	2211
	5452	C-T	T-M	51.11	13
	5460	G-A	A-T	4.44	2956
ND3	10,158	T-C	S-P	31.11	0
	10,191	T-C	S-P	17.78	0
	10,086	A-G	N-D	86.67	414
	10,237	T-C	I-T	100	68
	10,398	A-G	T-A	51.11	20,416
ND4	11,240	C-T	L-F	97.78	0
	11,696	G-A	V-I	6.67	275
	11,084	A-G	T-A	86.67	190
	11,232	T-C	L-P	93.33	0
	11,253	T-C	I-T	42.22	239
	11,874	C-A	T-N	77.78	0
	11,919	C-T	S-F	86.67	0
	11,994	C-T	T-I	31.11	0
	12,026	A-G	I-V	62.22	216
ND4L	10,663	T-C	V-A	88.89	1
	10,680	G-A	A-T	93.33	18
ND5	13,514	A-G	D-G	100	0
	13,528	A-G	T-A	40	38
	13,708	G-A	A-T	33.33	3262
	12,338	T-C	M-T	71.11	141
	12,361	A-G	T-A	13.33	252
	12,397	A-G	T-A	20	318
	12,622	G-A	V-I	82.22	10
	12,634	A-G	I-V	97.78	130
	12,811	T-C	Y-H	55.56	547
	13,094	T-C	V-A	100	1
	13,135	G-A	A-T	8.89	429
	13,511	A-T	K-M	100	0
	13,831	C-A	L-M	31.11	3
	13,849	A-C	N-H	62.22	0
	13,967	C-T	T-M	13.33	139
	14,063	T-C	I-T	13.33	24
	14,091	A-T	K-N	100	0
ND6	14,453	G-A	A-V	93.33	0
	14,459	G-A	A-V	88.89	3
	14,482	C-A	M-I	31.11	2
	14,487	T-C	M-V	97.78	0
	14,502	T-C	I-V	77.78	168
	14,596	A-T	I-M	84.44	0
	14,163	C-T	A-T	15.56	12
	14,279	G-A	S-L	46.67	6
	14,325	T-C	N-D	17.78	49
	14,319	T-C	N-D	40	55
	14,340	C-T	V-M	17.78	21
	14,439	G-A	P-S	95.56	0
	14,482	C-G	M-I	31.11	0

### Presence of Pathogenic Variants Within Non-human Species

The m.3308T>C variant was present in 100% of the mt-ND1 sequences from species of the *Pan* genus (Table 1). Two additional mt-ND1 variants, m.4205T>C and m.4232T>C corresponding to amino acid changes L > S and I > T, respectively, were present in the same species, but absent in the remaining species. Both are thought to be non-pathogenic in humans. The m.4205T>C variant is reported in 10/49 R8a haplogroup sequences, whereas m.4232T>C is reported in 13/15 haplogroup R30a1 sequences and 551/552 haplogroup L0d sequences (Benson et al. 2005). A single L0d sequence from human population data was shown to carry both the ‘pathogenic’ m.3308T>C and the non-pathogenic m.4232T>C (van Oven and Kayser 2009).

**Fig. 1:**
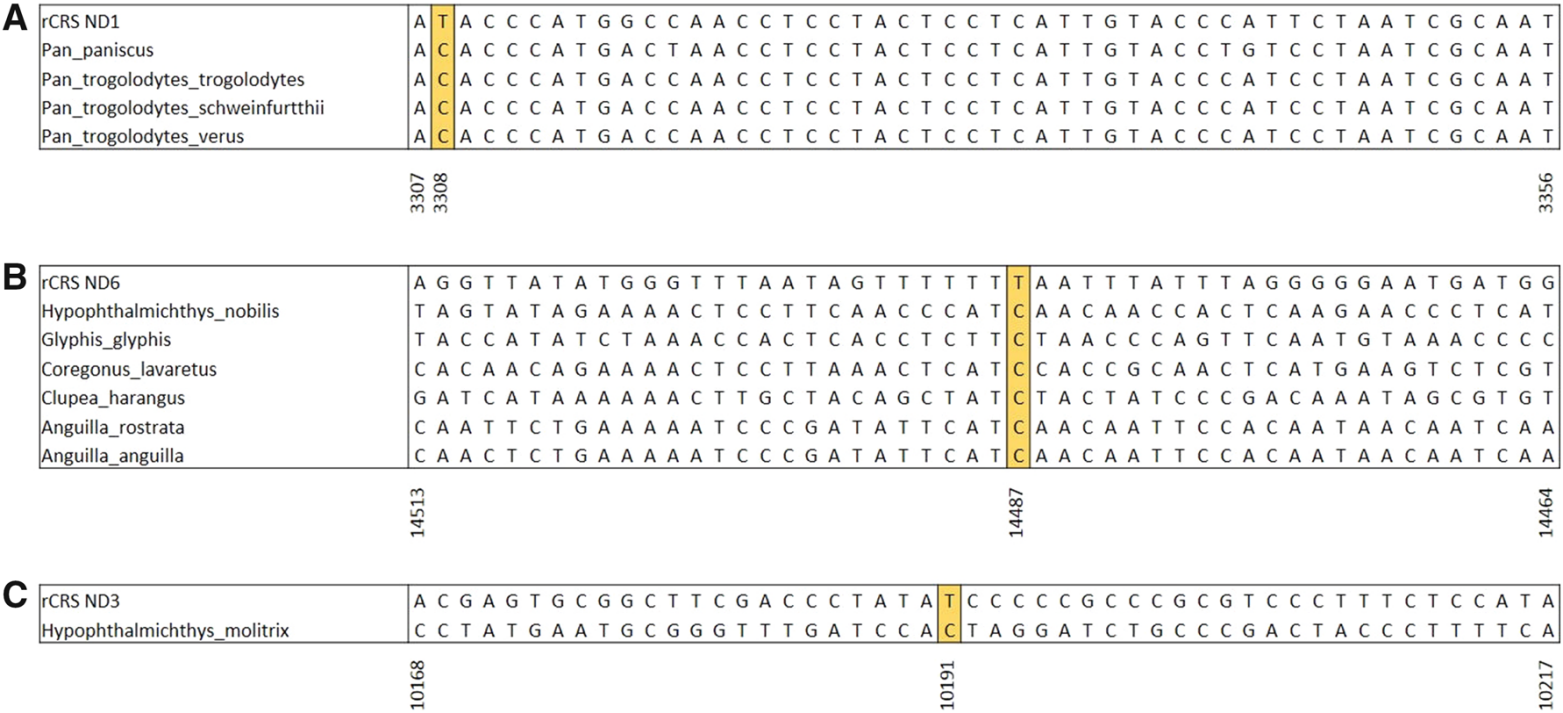
Consensus sequence alignments from species containing pathogenic variants. **a** Consensus sequences from the Pan genus demonstrating the presence of the 3308T>C variant. **b** Consensus sequences from species of fish demonstrating the presence of the 14487T>C variant. **c** Consensus sequences from *Hypophthalmichthys molitrix* demonstrating the presence of the 10191T>C variant. All consensus sequences were derived from the individual species multiple sequence alignments

The m.10191T>C mt-ND3 and m.14487T>C mt-ND6 human pathogenic variants were identified in non-mammalian Chordata, more specifically, species of fish (Table 1). The m.10191T>C variant was found in a single species, *Hypophthalmichthys molitrix*, while m.14487T>C was identified in six species. Again, the presence of these variants was seen in 100% of the sequence alignment from each of these species. However, although the m.10191T>C causes an S > P amino acid change in humans, an S > L amino acid change is seen in *Hypophthalmichthys molitrix* due to variation at all three bases of the codon. Similarly, the m.14487T>C variant that would produce an M > V change in humans, instead results in an M > G amino acid change in all six fish species in which it was identified, due to a second variation in the first base of the codon, see Fig. 1.

## Discussion

The results of this study focussing on the seven mtDNA encoded complex I genes differ from the previous study on the mt-tRNA genes (Queen et al. 2017) in which three definitely human pathogenic variants were identified in 100% of alignments in a single mt-tRNA gene, mt-tRNA-Leu (UUR), and a further two definitely pathogenic variants occurred as polymorphic variants in different species. Thus, five human definitely pathogenic variants of mt-tRNA-Leu (UUR) were either ubiquitous or common in other species. In contrast, across all seven mtDNA encoded complex I genes only three definitely pathogenic variants were found to be present in the non-human sequences; only one of which, m.3308T>C, presented its associated amino acid alteration in the non-human species. Prior work by Magalhães (2005) found 32 pathogenic variants in the tRNA genes and eight in the complex I genes (de Magalhaes 2005), this work being completed without the use of agreed algorithms to assign pathogenicity, as these were not agreed until after the completion of this path finding study.

Importantly, however, the pathogenicity of the m.3308T>C variant is contested in some contexts (Salas and Elson 2012). Such debates might be expected if a variant shows a deleterious effect on one lineage but not on another. m.3308T>C is a commonly found mt-ND1 polymorphism in some human lineages, which is generally perceived as strong evidence against a role in disease under the assumption of variants having the same effect in all lineage contexts. It is an African L1b and North American A2i haplogroup marker and is also evident in a small subset of L2a sequences (van Oven and Kayser 2009). However, it is rare in European sequences, appearing in 1/1063 J1c sequences and 1/443 T1a sequences in GenBank (van Oven and Kayser 2009) and has been associated with MELAS and sudden infant death syndrome in patients with European haplogroups (Opdal et al. 1999; Campos et al. 1997). This leads us to consider if “out of context” haplogroup variants should be considered as candidates for pathogenic changes. In some studies, such out of place haplogroup variants have been referred to as private changes (Herrnstadt et al. 2002).

Variation at m.3308T>C exchanges the initiation codon methionine for a threonine. In humans, an alternative initiation codon is found at the third amino acid (Opdal et al. 1999). It is thought that this truncation of two amino acids may influence the hydrophobicity of the protein N-terminal and its ability to anchor to the membrane (Campos et al. 1997). This variant was only present in species from the *Pan* genus (Table 1). Species containing m.3308T>C showed a methionine to threonine change and alternative initiation codon at position 3 as seen in humans (Opdal et al. 1999). The alignments revealed an L0d haplogroup marker, 4232T>C, present only in the non-human sequences containing the m.3308T>C variant. The L0d haplogroup is predominant within the South African Khoisan population. It is one of the deepest rooted haplogroups within the anatomically modern human mtDNA lineage (van Oven and Kayser 2009). A single L0d sequence containing both m.3308T>C and 4232T>C, with no reported disease association, is present on the GenBank database, EU092708.1.

Both m.10191T>C and m.14487T>C human pathogenic variants were conserved in species of fish (Table 1). Similarly, when studying mt-tRNA-Leu(UUR), three variants were found to be confined to species of fish, one of which was deemed pathogenic (Queen et al. 2017). In all these species of fish, further variation was seen within the codons containing these pathogenic variants. H*ypophthalmichthys molitrix* showed variation within the codon which may be sufficient to repress the penetrance of the m.10191T>C variant. Similarly, in species containing m.14487T>C, variation was seen at the first base of the codon, resulting in an M > G amino acid change rather than the M > V change seen in humans.

It is important to recall that the mtDNA complex 1 genes only account for seven of the 45 subunits that constitute complex I. Therefore, it is possible that nuclear variability contributes to the masking of pathogenic variants within the mt-protein-encoding genes in some contexts (van der Westhuizen et al. 2015). The interdependent nature of mitonuclear proteins suggests nuclear variability, particularly in the supernumerary subunits, could resolve stability within the protein complexes (Mimaki et al. 2012). The elegant study of Loewen and Ganetzky (2018) is an important exemplar when considering nuclear–mitochondrial interactions. Their paper showed that that the phenotypic severity of a complex 1 mutation causing Leigh syndrome phenotype varies depending on the mitochondrial background. Leigh syndrome is a severe disorder that is characterised by early, progressive neurodegeneration, with both intellectual and motor difficulties and deficient mitochondrial respiration (Lake et al. 2016).

In the current study, the pathogenicity variants were classified using accepted methodology as report (Mitchell et al. 2006), but it should be noted this method has not been reviewed in recent years. It is important to note the original method for the classification of mtDNA variants in the context of mt-tRNA mutations (McFarland et al. 2004) was found to be too conservative upon review (Yarham et al. 2012). Therefore, a review of the method presented by Mitchell et al. (2006) might be merited, particularly in light of a growing understanding of nuclear–mitochondrial interactions, as highlighted in recent works (Loewen and Ganetzky 2018), and with there being 19 variants that were classified as probably pathogenic in the species studied, see Table 1.

These data, along with the previous findings in mt-tRNA-Leu (UUR) (Queen et al. 2017), would suggest that sequence context is important in the expression and penetrance of mtDNA disease. However, it also suggests this phenomenon might be more important in mt-tRNA genes compared to mt-protein-encoding genes. This finding is perhaps linked to the differing effectiveness of purifying selection in the removal of deleterious or mildly deleterious variants in the protein-encoding genes and the mt-tRNA’s at the mitochondrial bottleneck (Stewart et al. 2008). Stewart et al. (2008) reported a rapid and strong elimination of nonsynonymous changes in protein-coding genes in a mouse model, which is the hallmark of purifying selection, but the removal of changes in the mt-tRNA and rRNA genes was much slower taking many more generations. The apparent tighter selective constraints on mtDNA protein-encoding genes is likely related to the multimeric nature of the mtDNA complexes and the need to maintain nuclear–mitochondrial compatibility (Mimaki et al. 2012). It might also be that the primary bottleneck occurs at a time when there are low levels of protein synthesis, but high respiratory demand, perhaps explaining the lack of efficacy of this selective process in the context of some pathogenic mt-tRNA mutations that are frequently transmitted in humans (Elson et al. 2009). It should also be noted that in adults presenting with disease resulting from mtDNA point mutations, mutations of the mt-tRNA’s are more common than those of the protein-encoding genes, with the m.3243A > G mutation being by far the most common (Gorman et al. 2015), while mutations of the complex 1 genes being frequently associated with severe paediatric disorders such a Leigh syndrome (Lake et al. 2016).

Nevertheless, this study has significant implications for diagnostic investigations in patients from understudied population groups as it supports the argument that there might not be a universal list of pathogenic variants for all lineages (van der Westhuizen et al. 2015). Together with the findings of Queen et al. (2017), it highlights the dangers of investigating variants in isolation without considering haplogroup context and assuming that what is true in one haplogroup context will be true in another. This work and that of others (Queen et al. 2017; Kern and Kondrashov 2004; Loewen and Ganetzky 2018) provides significant motivation for conducting sequencing or survey studies investigating the prevalence of common point mutations in understudied population groups, similar to that conducted previously in European populations (Elliott et al. 2008). Much work has been done in a number of Asian populations to better understand importance of sequence context, which has revealed population-specific mutations and lineage-specific effects (Ji et al. 2014; Zhang et al. 2012). In order to achieve comparable diagnostic abilities in all populations, additional studies are required to expand our knowledge of population variation in less studied populations, and gathering the laboratory based data to work-up mutations in individuals from these understudied populations. If it is demonstrated that haplogroup context is of greater importance in the expression and penetrance of mtDNA mutations than previously appreciated, then perhaps the role, if any, of mtDNA variation in complex traits might also be population specific.

## Electronic supplementary material

Below is the link to the electronic supplementary material.

Supplementary material 1 (DOCX 26 KB)
