# Supplementary material for: Haplogroup Context is Less Important in the Penetrance of Mitochondrial DNA Complex I Mutations Compared to mt-tRNA Mutations
Source: J Mol Evol. 2018 Jul 9;86(6):395–403. doi: 10.1007/s00239-018-9855-7 (PMC6061473; doi:10.1007/s00239-018-9855-7)
Supplement: Supplementary file 1 — Supplementary material 1 (DOCX 26 KB) [file 239_2018_9855_MOESM1_ESM.docx]

Journal of Molecular Evolution

Haplogroup context is less important in the penetrance of Mitochondrial DNA Complex I mutations compared to mt-tRNA mutations

Hannah O'Keefe^1,3^, Rachel A. Queen^1^, Surita Meldau^2,4^, Phillip Lord^3^, Joanna L. Elson^1,5 *^

1. Institute of Genetic Medicine, Newcastle University, Newcastle-upon-Tyne, NE1 3BZ, United Kingdom
2. Inherited Metabolic Disease Laboratory, National Health Laboratory Services, Cape Town, South Africa
3. School of Computing, Newcastle University, Newcastle-upon-Tyne, NE4 5TG, United Kingdom
4. Division of Chemical Pathology, University of Cape Town, Cape Town, South Africa
5. Centre for Human Metabonomics, North-West University, Potchefstroom, South Africa

*Correspondence should be sent to: Dr. Joanna Elson, Institute of Genetic Medicine, Newcastle University, Newcastle-upon-Tyne, NE1 3BZ, United Kingdom; Email: joanna.elson@newcastle.ac.uk

| **Species** | **Common Name** | **Number of Sequences before QC** | **Number of Sequences after QC** |
| --- | --- | --- | --- |
| *Anguilla anguilla* | European Eel | 55 | 54 |
| *Anguilla rostrata* | American Eel | 51 | 51 |
| *Balaenoptera physalus* | Fin Whale | 154 | 154 |
| *Bison bison* | Bison | 34 | 34 |
| *Bos grunniens* | Yak | 83 | 83 |
| *Bos taurus* | Cow | 275 | 275 |
| *Canis lupus familiaris* | Dog | 391 | 391 |
| *Clupea harengus* | Alantic Herring | 100 | 100 |
| *Coregonus lavaretus* | European whitefish | 81 | 80 |
| *Equus caballus* | Horse | 254 | 245 |
| *Gallus gallus* | Red Jungle Fowl | 66 | 66 |
| *Glyphis glyphis* | Speartooth Shark | 94 | 94 |
| *Hypophthalmichthys molitrix* | Silver carp | 30 | 29 |
| *Hypophthalmichthys nobilis* | Bighead carp | 36 | 35 |
| *Macaca fascicularis* | Crab-eating macaque | 44 | 44 |
| *Mus musculus* | mouse | 53 | 50 |
| *Mus musculus domesticus* | House mouse | 59 | 59 |
| *Myodes glareolus* | Bank vole | 35 | 35 |
| *Orcinus orca* | Killer Whale | 87 | 87 |
| *Ovis aries* | Sheep | 94 | 94 |
| *Pan paniscus* | Banobo | 54 | 54 |
| *Pan troglodytes schweinfurthii* | Eastern chimpanzee | 33 | 33 |
| *Pan troglodytes troglodytes* | Central chimpanzee | 56 | 54 |
| *Pan troglodytes verus* | Western chimpanzee | 30 | 30 |
| *Rattus norvegicus* | Brown Rat | 66 | 66 |
| *Sus scrofa* | Wild Boar | 150 | 150 |
| *Syncerus caffer* | African buffalo | 45 | 45 |
| *Tursiops truncatus* | Common bottlenose Dolphin | 50 | 50 |
| *Urocyon littoralis catalinae* | Island Fox | 41 | 41 |
| *Urocyon littoralis clementae* | Island Fox | 33 | 33 |
| *Urocyon littoralis santacruzae* | Island Fox | 42 | 42 |
| *Ursus arctos* | Brown Bear | 74 | 74 |
| *Ursus spelaeus* | Cave Bear (extinct) | 34 | 20 |

Supplementary Table 1: The 33 chordate species with greater than 30 complete mtDNA sequences used in the initial phase of the study

| Gene | Position | Variant | Disease Association | Status | Species |
| --- | --- | --- | --- | --- | --- |
| ND1 | **3316** | **G-A** | **Diabetes, LHON, Progressive external ophthalmoplegia** | **Neutral** | ***Pan paniscus, Pan trogolodytes trogolodytes, Pan trogolodytes schweinfurthii, Pan trogolodytes verus, Macaca Fascicularis, Mus Musculus, Mus Musculus Domesticus, Rattus Norvegicus, Myodes glareolus, Anguilla Anguilla, Anguilla Rostrata, Bos taurus, Bos grunniens, Ovis aries, Clupea harengus, Coregonus Lavaretus, Hypophthalmichthys molitrix, Hypophthalmichthys nobilis, Balaenoptera physalus, Bison bison, Orcinus orca, Sus scrofa, Syncerus caffer, Tursiops truncatus, Canis lupis familiaris, Ursus arctos, Ursus spelaeus*** |
|  | **3337** | **G-A** | **Cardiomyopathy** | **Possibly pathogenic** | ***Macaca Fascicularis, Mus Musculus, Rattus Norvegicus, Bos taurus, Bos grunniens, Ovis aries, Clupea harengus, Coregonus Lavaretus, Gallus Gallus, Glyphis Glyphis, Hypophthalmichthys molitrix, Bison bison, Sus scrofa, Syncerus caffer, Canis lupis familiaris, Urocyon litoralis catalinae, Urocyon litoralis clememtae, Urocyon litoralis santacruzae*** |
|  | **3340** | **T-C** | **Encephaloneuromyopathy** | **Possibly pathogenic** | ***Macaca Fascicularis*** |
|  | **3397** | **A-G** | **Alzheimers disease, parkinsons disease, possible left ventricular non compaction** | **Neutral** | ***Gallus Gallus*** |
|  | **3421** | **G-A** | **Maternally inherited diabetes and deafness** | **Neutral** | ***Pan paniscus, Pan trogolodytes trogolodytes, Pan trogolodytes schweinfurthii, Pan trogolodytes verus, Macaca Fascicularis, Mus Musculus, Mus Musculus Domesticus, Rattus Norvegicus, Myodes glareolus, Anguilla Anguilla, Anguilla Rostrata, Coregonus Lavaretus, Equus caballus, Glyphis Glyphis, Balaenoptera physalus, Orcinus orca, Tursiops truncatus, Canis lupis familiaris, Urocyon litoralis catalinae, Urocyon litoralis clememtae, Urocyon litoralis santacruzae, Ursus arctos, Ursus spelaeus*** |
|  | **3496** | **G-T** | **LHON** | **Neutral** | ***Pan paniscus, Pan trogolodytes trogolodytes, Pan trogolodytes schweinfurthii, Pan trogolodytes verus, Macaca Fascicularis, Mus Musculus, Mus Musculus Domesticus, Myodes glareolus, Glyphis Glyphis, Hypophthalmichthys molitrix*** |
|  | **3497** | **C-T** | **LHON** | **Neutral** | ***Mus Musculus, Mus Musculus Domesticus, Rattus Norvegicus, Myodes glareolus, Equus caballus, Gallus Gallus, Canis lupis familiaris, Urocyon litoralis catalinae, Urocyon litoralis clememtae, Urocyon litoralis santacruzae, Ursus arctos, Ursus spelaeus*** |
|  | **3644** | **T-C** | **Bipolar associated** | **Possibly pathogenic** | ***Coregonus Lavaretus*** |
|  | **3700** | **G-A** | **LHON** | **Possibly pathogenic** | ***Coregonus Lavaretus*** |
|  | **3736** | **G-A** | **LHON** | **Neutral** | ***Pan trogolodytes trogolodytes*** |
|  | **3745** | **G-A** | **Adaptive high altitude** | **Neutral** | ***Macaca Fascicularis*** |
|  | **3796** | **A-G** | **Adult on-set dystonia** | **Possibly pathogenic** | ***Pan trogolodytes trogolodytes, Anguilla Anguilla, Anguilla Rostrata, Clupea harengus, Coregonus Lavaretus, Glyphis Glyphis, Hypophthalmichthys molitrix, Hypophthalmichthys nobilis*** |
|  | **3833** | **T-A** | **Pseudoexfoliation Glaucoma** | **Neutral** | ***Clupea harengus, Glyphis Glyphis*** |
|  | **3866** | **T-C** | **LHON and Limb claudation** | **Possibly pathogenic** | ***Macaca Fascicularis, Clupea harengus, Bison bison*** |
|  | **4132** | **G-A** | **NAION associated** | **Neutral** | ***Pan paniscus, Macaca Fascicularis*** |
|  | **4142** | **G-A** | **Developmentl delay, seizure, hypotonia** | **Possibly pathogenic** | ***Sus scrofa*** |
| ND2 | **4648** | **T-C** | **Pseudoexfoliation Glaucoma** | **Neutral** | ***Sus scrofa*** |
|  | **4833** | **A-G** | **Glaucoma** | **Possibly pathogenic** | ***Clupea harengus, Balaenoptera physalus, Tursiops truncatus, Canis lupis familiaris, Ursus arctos, Ursus spelaeus*** |
|  | **4917** | **A-G** | **LHON, Insulin resistance, AMD, NRTI-PN** | **Possibly pathogenic** | ***Macaca Fascicularis, Myodes glareolus, Anguilla Anguilla, Anguilla Rostrata, Hypophthalmichthys molitrix, Hypophthalmichthys nobilis*** |
|  | **5178** | **C-A** | **Longevity, extraversion MI, AMS protection, blood iron metabolism** | **Neutral** | ***Mus Musculus, Mus Musculus Domesticus, Rattus Norvegicus, Myodes glareolus, Bos taurus, Bos grunniens, Equus caballus, Gallus Gallus, Hypophthalmichthys molitrix, Hypophthalmichthys nobilis, Balaenoptera physalus, Bison bison, Orcinus orca, Sus scrofa, Syncerus caffer, Tursiops truncatus*** |
|  | **5452** | **C-T** | **LHON** | **Neutral** | ***Anguilla Anguilla, Anguilla Rostrata, Coregonus Lavaretus, Equus caballus, Gallus Gallus, Glyphis Glyphis, Hypophthalmichthys molitrix, Hypophthalmichthys nobilis*** |
|  | **5460** | **G-A** | **Alzheimers disease, Parkinsons disease** | **Possibly pathogenic** | ***Pan paniscus, Pan trogolodytes trogolodytes, Pan trogolodytes schweinfurthii, Pan trogolodytes verus, Macaca Fascicularis, Mus Musculus, Mus Musculus Domesticus, Rattus Norvegicus, Myodes glareolus, Clupea harengus, Coregonus Lavaretus, Equus caballus, Glyphis Glyphis, Hypophthalmichthys molitrix, Hypophthalmichthys nobilis, Canis lupis familiaris, Urocyon litoralis catalinae, Urocyon litoralis clememtae, Urocyon litoralis santacruzae*** |
| ND3 | **10086** | **A-G** | **Hypersensitive end-stage renal disease** | **Neutral** | ***Equus caballus*** |
|  | **10237** | **T-C** | **LHON** | **Neutral** | ***Anguilla Anguilla, Anguilla Rostrata, Clupea harengus, Coregonus Lavaretus, Glyphis Glyphis, Hypophthalmichthys molitrix, Hypophthalmichthys nobilis, Urocyon litoralis catalinae, Ursus spelaeus*** |
|  | **10398** | **A-G** | **Parkinsons disease protective factor, longevity, altered cell pH, metabolic syndrome, breast cancer risk, ADHD** | **Possibly pathogenic** | ***Pan paniscus, Pan trogolodytes trogolodytes, Pan trogolodytes schweinfurthii, Macaca Fascicularis, Anguilla Anguilla, Clupea harengus, Gallus Gallus, Balaenoptera physalus, Sus scrofa, Tursiops truncatus, Canis lupis familiaris, Ursus arctos, Ursus spelaeus*** |
| ND4 | **11084** | **A-G** | **Alzheimers disease, Parkinsons disease, MELAS** | **Neutral** | ***Anguilla Anguilla, Anguilla Rostrata, Gallus Gallus, Glyphis Glyphis*** |
|  | **11232** | **T-C** | **Chronic progressive external ophthalmoplegia** | **Possibly pathogenic** | ***Anguilla Anguilla, Anguilla Rostrata*** |
|  | **11253** | **T-C** | **LHON, Parkinsons disease** | **Neutral** | ***Pan paniscus, Pan trogolodytes trogolodytes, Bos taurus, Bos grunniens, Orcinus orca, Syncerus caffer, Tursiops truncatus*** |
|  | **11874** | **C-A** | **LHON** | **Neutral** | ***Rattus Norvegicus*** |
|  | **11919** | **C-T** | **Thyroid cancer cell lines** | **Possibly pathogenic** | ***Anguilla Anguilla, Anguilla Rostrata, Clupea harengus, Coregonus Lavaretus, Hypophthalmichthys molitrix, Hypophthalmichthys nobilis, Balaenoptera physalus, Ursus spelaeus*** |
|  | **11994** | **C-T** | **Oligoasthenoteratozoospermia** | **Neutral** | ***Macaca Fascicularis, Mus Musculus, Mus Musculus Domesticus, Rattus Norvegicus, Myodes glareolus, Anguilla Anguilla, Anguilla Rostrata, Bos grunniens, Ovis aries, Clupea harengus, Coregonus Lavaretus, Equus caballus, Gallus Gallus, Glyphis Glyphis, Hypophthalmichthys molitrix, Hypophthalmichthys nobilis, Balaenoptera physalus, Bison bison, Orcinus orca, Sus scrofa, Syncerus caffer, Tursiops truncatus, Canis lupis familiaris, Urocyon litoralis catalinae, Urocyon litoralis clememtae, Urocyon litoralis santacruzae, Ursus arctos*** |
|  | **12026** | **A-G** | **Diabetes mellitus** | **Neutral** | ***Urocyon litoralis catalinae, Urocyon litoralis clememtae, Urocyon litoralis santacruzae, Ursus arctos, Ursus spelaeus*** |
| ND4L | **10680** | **G-A** | **LHON** | **Possibly pathogenic** | ***Macaca Fascicularis, Coregonus Lavaretus, Gallus Gallus*** |
| ND5 | **12338** | **T-C** | **Deafness, LHON** | **Neutral** | ***Pan paniscus*** |
|  | **12361** | **A-G** | **Non alcoholic fatty liver disease** | **Neutral** | ***Pan trogolodytes trogolodytes, Urocyon litoralis catalinae, Urocyon litoralis clememtae, Urocyon litoralis santacruzae*** |
|  | **12397** | **A-G** | **Early on-set Parkinsons disease** | **Possibly pathogenic** | ***Pan trogolodytes trogolodytes, Pan trogolodytes schweinfurthii, Pan trogolodytes verus, Macaca Fascicularis, Bos grunniens, Ovis aries, Orcinus orca, Tursiops truncatus*** |
|  | **12622** | **G-A** | **Leigh disease** | **Possibly pathogenic** | ***Macaca Fascicularis, Clupea harengus, Balaenoptera physalus, Orcinus orca,Tursiops truncatus, Canis lupis familiaris*** |
|  | **12634** | **A-G** | **Thyroid cancer cell lines** | **Possibly pathogenic** | ***Clupea harengus*** |
|  | **12811** | **T-C** | **LHON** | **Possibly pathogenic** | ***Macaca Fascicularis, Bison bison*** |
|  | **13094** | **T-C** | **Ataxia + PEO, MELAS, Myoclonus fatigue** | **Possibly pathogenic** | ***Ursus spelaeus*** |
|  | **13135** | **G-A** | **Hypertrophic cardiomyopathy susceptibility** | **Possibly pathogenic** | ***Macaca Fascicularis, Mus Musculus, Mus Musculus Domesticus, Rattus Norvegicus, Myodes glareolus, Anguilla Anguilla, Anguilla Rostrata, Bos taurus, Bos grunniens, Ovis aries, Clupea harengus, Coregonus Lavaretus, Equus caballus, Hypophthalmichthys molitrix, Hypophthalmichthys nobilis, Balaenoptera physalus, Bison bison, Orcinus orca, Sus scrofa, Syncerus caffer, Tursiops truncatus, Canis lupis familiaris, Urocyon litoralis catalinae, Urocyon litoralis clememtae, Urocyon litoralis santacruzae, Ursus arctos, Ursus spelaeus*** |
|  | **13511** | **A-T** | **Leigh-like syndrome** | **Possibly pathogenic** | ***Coregonus Lavaretus*** |
|  | **13831** | **C-A** | **Thyroid cancer cell lines** | **Possibly pathogenic** | ***Bos taurus, Bos grunniens, Ovis aries, Clupea harengus, Equus caballus, Gallus Gallus, Balaenoptera physalus, Bison bison, Sus scrofa, Syncerus caffer, Canis lupis familiaris, Urocyon litoralis catalinae, Urocyon litoralis clememtae, Urocyon litoralis santacruzae, Ursus arctos, Ursus spelaeus*** |
|  | **13849** | **A-C** | **MELAS** | **Neutral** | ***Coregonus Lavaretus, Glyphis Glyphis*** |
|  | **13967** | **C-T** | **LHON** | **Neutral** | ***Macaca Fascicularis, Mus Musculus, Mus Musculus Domesticus, Anguilla Rostrata, Bos taurus, Bos grunniens, Ovis aries, Balaenoptera physalus, Bison bison, Sus scrofa, Syncerus caffer, Tursiops truncatus, Canis lupis familiaris, Urocyon litoralis catalinae, Urocyon litoralis clememtae, Urocyon litoralis santacruzae, Ursus arctos, Ursus spelaeus*** |
|  | **14063** | **T-C** | **LHON** | **Neutral** | ***Pan paniscus, Pan trogolodytes trogolodytes, Pan trogolodytes schweinfurthii, Pan trogolodytes verus, Macaca Fascicularis, Anguilla Anguilla, Anguilla Rostrata, Ovis aries, Coregonus Lavaretus, Equus caballus, Glyphis Glyphis, Ursus arctos, Ursus spelaeus*** |
|  | **14091** | **A-T** | **Developmental delay, seizure, hearing loss, diabetes** | **Possibly pathogenic** | ***Anguilla Rostrata, Clupea harengus, Equus caballus, Gallus Gallus, Hypophthalmichthys molitrix*** |
| ND6 | **14163** | **C-T** | **Deafness** | **Neutral** | ***Mus Musculus Domesticus, Rattus Norvegicus, Myodes glareolus, Bos taurus, Bos grunniens, Ovis aries, Equus caballus, Gallus Gallus, Balaenoptera physalus, Bison bison, Orcinus orca, Syncerus caffer, Tursiops truncatus, Canis lupis familiaris, Urocyon litoralis catalinae, Urocyon litoralis clememtae, Urocyon litoralis santacruzae, Ursus arctos, Ursus spelaeus*** |
|  | **14279** | **G-A** | **LHON** | **Neutral** | ***Mus Musculus Mus, Rattus Norvegicus, Myodes glareolus, Bos taurus, Glyphis Glyphis*** |
|  | **14325** | **T-C** | **LHON** | **Neutral** | ***Macaca Fascicularis, Mus Musculus, Rattus Norvegicus, Myodes glareolus, Bos taurus, Bos grunniens, Coregonus Lavaretus, Equus caballus, Gallus Gallus, Glyphis Glyphis, Hypophthalmichthys molitrix, Hypophthalmichthys nobilis, Balaenoptera physalus, Bison bison, Orcinus orca, Syncerus caffer, Tursiops truncatus*** |
|  | **14319** | **T-C** | **Early on-set Parkinsons disease** | **Possibly pathogenic** | ***Pan trogolodytes verus, Rattus Norvegicus, Anguilla Anguilla, Anguilla Rostrata, Clupea harengus, Coregonus Lavaretus, Gallus Gallus, Glyphis Glyphis, Hypophthalmichthys molitrix, Hypophthalmichthys nobilis, Balaenoptera physalus*** |
|  | **14340** | **C-T** | **Sensorineural hearing loss** | **Neutral** | ***Pan trogolodytes trogolodytes, Pan trogolodytes schweinfurthii, Pan trogolodytes verus, Rattus Norvegicus, Clupea harengus*** |
|  | **14439** | **G-A** | **Mitochondrial respiratory chain disorder** | **Possibly pathogenic** | ***Anguilla Anguilla, Anguilla Rostrata, Clupea harengus, Coregonus Lavaretus, Glyphis Glyphis, Hypophthalmichthys molitrix, Hypophthalmichthys nobilis*** |
|  | **14482** | **C-G** | **LHON** | **Possibly pathogenic** | ***Urocyon litoralis catalinae, Urocyon litoralis santacruzae, Ursus arctos*** |

Supplemental Table 2. Possibly pathogenic or Neutral human mitochondrial variants present in the alignments of one or more chordate species across all 7 complex I genes. Disease association is listed in accordance with reports from the MitoMap online database [Accessed:02-2017].
